# Pineapple (*Ananas comosus* L.) By-Products Valorization: Novel Bio Ingredients for Functional Foods

**DOI:** 10.3390/molecules26113216

**Published:** 2021-05-27

**Authors:** Diana I. Santos, Cátia F. Martins, Renata A. Amaral, Luísa Brito, Jorge A. Saraiva, António A. Vicente, Margarida Moldão-Martins

**Affiliations:** 1 LEAF, Linking Landscape, Environment, Agriculture and Food, School of Agriculture, University of Lisbon, Tapada da Ajuda, 1349-017 Lisbon, Portugal; dianaisasantos@isa.ulisboa.pt (D.I.S.); catiamartins@isa.ulisboa.pt (C.F.M.); lbrito@isa.ulisboa.pt (L.B.); 2 LAQV-REQUIMTE, Department of Chemistry, University of Aveiro, 3810-193 Aveiro, Portugal; renata.amaral@ua.pt (R.A.A.); jorgesaraiva@ua.pt (J.A.S.); 3 CEB, Centro de Engenharia Biológica, Departamento de Engenharia Biológica, Universidade de Minho, Campus de Gualtar, 4710-057 Braga, Portugal; avicente@deb.uminho.pt

**Keywords:** pineapple by-products, bioactive compounds, novel food ingredient, sustainability

## Abstract

Pineapple is consumed on a large scale around the world due to its appreciated sensorial characteristics. The industry of minimally processed pineapple produces enormous quantities of by-products (30–50%) which are generally undervalued. The end-of-life of pineapple by-products (PBP) can be replaced by reuse and renewal flows in an integrated process to promote economic growth by reducing consumption of natural resources and diminishing food waste. In our study, pineapple shell (PS) and pineapple core (PC), vacuum-packed separately, were subjected to moderate hydrostatic pressure (225 MPa, 8.5 min) (MHP) as abiotic stress to increase bromelain activity and antioxidant capacity. Pressurized and raw PBP were lyophilized to produce a stable powder. The dehydrated samples were characterized by the following methodologies: chemical and physical characterization, total phenolic compounds (TPC), antioxidant capacity, bromelain activity, microbiology, and mycotoxins. Results demonstrated that PBP are naturally rich in carbohydrates (66–88%), insoluble (16–28%) and soluble (2–4%) fiber, and minerals (4–5%). MHP was demonstrated to be beneficial in improving TPC (2–4%), antioxidant activity (2–6%), and bromelain activity (6–32%) without affecting the nutritional value. Furthermore, microbial and mycotoxical analysis demonstrated that powdered PC is a safe by-product. PS application is possible but requires previous decontamination to reduce the microbiological load.

## 1. Introduction

Pineapple has potential in the fresh-cut form market due to its appreciated sensorial characteristics (flavor, juiciness, and taste) and the demand for immediate consumption [1]. In addition to its sensorial quality, the nutritional properties of pineapple also deserve interest since it is a good source of phenolics, and consequently, it is rich in antioxidant activity [2].

The consumption of pineapple has health benefits, namely on the digestive system, and helps to maintain a balanced diet [3]. Pineapple has essential minerals such as manganese with important functions in bone formation and enzyme activation, and copper with functions on iron absorption, blood pressure regulation, and heart rate [4]. Pineapple is a source of a mixture of several proteases, namely stem bromelain and fruit bromelain [5,6].

Bromelain is an enzyme naturally present in pineapple by-products (PBP; shell, core, crown, and leaves) that has numerous advantages in the digestive and cardiovascular systems, along with an anti-inflammatory effect, anticancer, and antimicrobial agent [7]. These characteristics, associated with the high content of phenolic compounds and antioxidant activity, are an added value for health [8]. For that reason, bromelain has been used in several areas such as food, pharmaceuticals, cosmetics, and other industries. It may have several industrial food applications, notably in beer clarification, meat and fish brewing (meat tenderizing), baking cookies, grain protein solubilization, functional protein pre-digestion, and protein hydrolysate production [9,10].

Moderate hydrostatic pressure treatments (225 MPa, 8.5 min) are applied on pineapple by-products such as abiotic stress, activate cellular processes, and enhancing the accumulation of bromelain and phenolic compounds with antioxidant activity [11]. Dehydrated pineapple by-product, enriched in bromelain using hydrostatic pressure treatment has been used in marinated beef (10–20 mg tyrosine.100 g^−1^ meat), resulting in a 41% reduction of beef hardness, 8% reduced pH (5.44 to 4.99), and increased marination yield (4%) [12]. In the pharmaceutical industry, it is used to facilitate digestion, tumor growth modulation, third-degree burns, enhancing antibiotic action, and as a medication for the oral systemic treatment of inflammation, blood coagulation associated, and cancerous disorders [13]. Bromelain is also used in the cosmetics industry as an active ingredient in facial and body care products to provide smooth cell peeling [10].

Fruit by-products produced during processing (shell, core, stem, and crown), representing approximately 50% (*w*/*w*) of the total weight of the pineapple, are used mainly in animal feed and in the pharmaceutical industry. Pineapple residues are distributed in 29–40% shell, 9–10% core, 2–6% stem, and 2–4% crown and have the potential to be transformed into value-added products [14,15]. The by-products contain compounds such as dietary fiber, vitamins, minerals, phenolic compounds, and other bioactive compounds. Taking into account the benefits mentioned above, PBP are recommended for incorporation into human food [4,14,16]. The use of agro-industrial by-products is very important due to the large amount of material available, low cost, and characteristics that allow extracting/obtaining added-value compounds [17]. The waste generated by the food industry is often a loss of valuable materials that, if treated as by-products, have the potential to be used in other productions and thus reduce management problems (economic and environmental) [18]. The process for recovering by-products must include food safety to ensure the application of the ingredients in the food industry. Therefore, what once was waste can be valued in an integrated way and become a secondary raw material [19,20].

PBP are sensitive to microbial deterioration, entail costs in their disposal, and cause environmental problems. The use of these by-products would be an excellent opportunity to innovate (develop new products) and reduce the amount of waste caused by the food industry [14].

The present study, within the scope of the circular economy concept, aims to: (i) contribute to the reduction of food waste through the valorization of PBP (shell and core); (ii) to study the effect of abiotic stress applied by hydrostatic pressure on the composition of PBP, namely antioxidant capacity and bromelain activity, and microbial and toxicological quality.

## 2. Results and Discussion

### 2.1. Color Measurement

The analysis of the values of the CIE L*a*b* color parameters showed that the samples of the dehydrated pineapple by-product, core, and shell, presented a similar color, in the yellow/green range, with the color of the pineapple core (PC) being lighter (higher significant values of L*) (Table 1). The photos make it possible to observe differences between the lightness of the core and shell pineapple samples. The color of the samples is quite neutral, and it is expected that the addition to food will not have very marked changes. The samples did not show visible differences between pressurized and non-pressurized samples, although the color evaluation identified a slight variation in the parameter a* of the PS and in the parameter b* in the PC. The visual characteristics of a product have a strong impact on the consumer’s first opinion. Color is a very important attribute in sensory analysis since it induces the consumer to expect a certain taste or expectation, in addition to influencing perceptions regarding the remaining sensory attributes.

### 2.2. Chemical and Physical Characterization

The chemical and physical characterization of the PS and core samples is resumed in Table 2. The global analysis of the data makes it possible to observe differences between the core and shell pineapple samples, however, the effect of the hydrostatic pressure did not affect the basic composition of the PBP samples. The results of the samples of raw and pressurized PBP are not presented, since the values were similar.

PS samples presented lower moisture (5.50 ± 0.28%) than PC (7.96 ± 0.17%). However, all samples had similar and low values of a_w_ (0.264–0.299) to allow good preservation characteristics. Higher moisture values in the PC and similar values of a_w_ can be justified by the higher sugar content that promotes greater water retention. Megías-Pérez et al. (2014) [21] studied pineapple dehydrated fruit and obtained similar results (10.4% moisture and a_w_ of 0.532) to those obtained for the PC by-products.

The caloric value of PS and PC samples (374.52 and 363.89 kcal.100 g^−1^ dry matter) is higher than that of some dehydrated fruits such as raisins, apples, figs, peaches, pears, and plums (347–357 kcal.100 g^−1^ dry matter) [22]. However, it is lower than the value mentioned by previous authors for nuts (553–718 kcal.100 g^−1^) and for cereals (corn, oats, and barley (~425.22 kcal.100 g^−1^) [23].

As observed for most dehydrated fruits, studied PBP samples presented low protein content (<3.86%). The values observed in the shell (3.86%) were significantly higher than those observed in the core (2.71%). Other studies that performed the determination of protein in PBP obtained similar results to the present study. The protein content determined in pineapple pomace was 4.71% [24], in pineapple co-product (shell and core) was 4% [25], and in PS was 4.53% [26] and 4.10% [27].

As expected, PC samples presented significantly higher values of the total carbohydrates (87.58%) than PS samples (66.44%), contrary to that verified for the dietary fiber content. Kadam et al. (2012) [28] have also found high values for total carbohydrates of 41.98 to 65.60% for dry pineapple powder and Megías-Pérez et al. (2014) [21] determined 76.8 ± 0.9% (g.100 g^−1^ dry matter) of total sugar content in dehydrated pineapple samples. The studies with dry pineapple powder and dehydrated pineapple samples are in accordance with the results obtained for PBP.

PC samples presented lower values of dietary fiber (17.70%) compared to PS samples (32.42%) (Table 2). The fraction of insoluble fiber was the most representative (28.82 and 16.17% for PC and shell, respectively). It should be noted that despite being the least representative, the fraction of soluble fiber (1.53–3.63) was relatively high compared to other fruits or cereals. This may be important as dietary fiber is important to reduce the blood glucose of the consumers. Campos et al. (2020) [29] conducted a study with the solid fraction (press cake) of PS and obtained 38.30% insoluble fiber and 7.99% soluble fiber, and regarding PC, obtained 44.37% insoluble fiber and 4.57% soluble fiber. The determination of dietary fiber in pineapple pomace found 45.22% total dietary fiber, 44.44% insoluble fiber, and 0.78% soluble fiber [24]. The pineapple co-products showed 75.8% total dietary fiber, 75.2% insoluble fiber, and 0.6% soluble fiber [25]. Our results for PBP were according to other authors, although the studied samples were not exactly the same. This study evaluated the PS and core independently and without any extraction or concentration. Analyzing the results, it can be assured that the by-products present considerable values of dietary fiber, most of which are insoluble fibers, as obtained in other studies [24,25,29]. The PS contains a considerable amount of insoluble fiber that involves mainly cellulose, substances from pectin, hemicellulose, and lignin [30].

Regarding the effect of hydrostatic pressure, in this study (225 MPa), it was not so evident to that observed by Wennberg and Nyman (2004) [31] in the study of the effect of hydrostatic pressure (400 and 500 MPa combined with temperature 20, 50 and 80 °C) in white cabbage (*Brassica oleracea*). Wennberg and Nyman (2004) [31] concluded that the total dietary fiber content of white cabbage was slightly affected by the pressure treatment, but considerable effects were observed on the results of the soluble and insoluble fibers. The proportion of soluble fiber was reduced at all temperatures to pressures of 400 MPa. The development of new foods products with specific health benefits may be based on increasing the solubility of dietary fiber improved by the pressure treatment and temperature. 

The PS samples showed an ash content (4.23 ± 0.13 g.100 g^−1^ dry matter) smaller than the PC samples (4.83 ± 0.28 g.100 g^−1^ dry matter) (Table 2). Hydrostatic pressure treatment did not influence the ash-based composition and mineral content, and the results are not shown. The values obtained for ash in other studies with pineapple waste samples showed a wide range of values (1–5%) [27,30,32]. The ash values obtained in other studies with PS corroborate the values obtained in the present study: 4.81% [30], 4.7% [33], and 4.14% [34]. A study carried out recently obtained values of 6.8% (dry matter basis) in samples of PS [26], which confirmed the great variability of results obtained in this pineapple constituent. One study with PC samples showed 1.3% of ash with 10% moisture [35], which indicates approximately 1.44% (dry matter basis), a lower value than that was observed in the present study. The characterization of the chemical composition is still little studied and further studies need to be carried out, especially in PC samples.

The samples of PBP showed a group of minerals (Table 2) of interest for human health. The PC samples showed a higher amount of potassium and magnesium compared to the PS (*p* < 0.05), while the PS samples showed a higher amount in the remaining analyzed minerals. The minerals present in pineapple juice powder in greater quantities are sodium, potassium, calcium, magnesium, and phosphorus, which is in agreement with the obtained results in the present study, except for sodium [36]. The PBP, as well as the fruits, also have interesting amounts of minerals. The quantification of minerals in PS defined calcium and potassium as the majority, although it presents a number of other minerals (bromine, cobalt, chromium, cesium, iron, lanthanum, sodium, rubidium, scandium, and zinc) in their composition [37]. The PC presents calcium, sodium, potassium, and magnesium in greater amounts and iron and zinc in minor amounts [38].

### 2.3. Total Phenolic Content and Antioxidant Activity (DPPH, FRAP, and ABTS)

**Figure 1:**
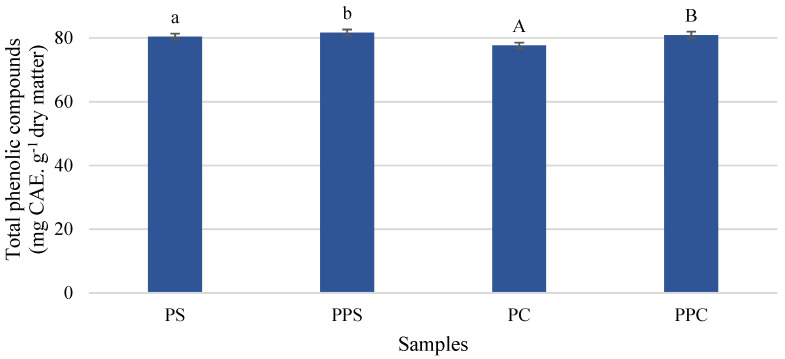
Total phenolic content in pineapple shell (PS) and pineapple core (PC) samples before and after pressurization (shell, PPS; core, PPC). Error bars represent ± standard deviation (n = 9). Different letters express significant differences between pineapple shell (lowercase letters) and pineapple core samples (capital letters).

The total phenolic content (Figure 1) showed higher values in pressurized samples (shell: 81.67 mg CAE·g^−1^ dry matter; core: 80.89 mg CAE·g^−1^ dry matter) compared to non-pressurized samples (shell: 80.42 mg CAE. dry g^−1^ dry matter; core: 77.61 mg CAE·g^−1^ dry matter). The hydrostatic pressure promoted a significant increase (*p* < 0.05) of 2 and 4% in the pineapple shell and pineapple core samples, respectively. The increase in the phenolic content due to pressurization was also verified by Santos et al. (2020) [11], but at different levels. The time between fresh fruit cutting and pressure processing is an important factor, and further studies on this subject should be developed since the longer storage time between cutting and pressure processing showed higher levels of total phenolic compounds [39].

The values of total phenolic content are commonly expressed in gallic acid, but in the present work, they are expressed in chlorogenic acid since it is the major phenolic compound in pineapple samples [40]. The phenolic content in the PBP reported by Campos et al. (2020) [29] was 1.31 mg·L^−1^ in core juice and 0.85 mg·L^−1^ in shell juice, expressed in chlorogenic acid equivalent.

**Figure 2:**
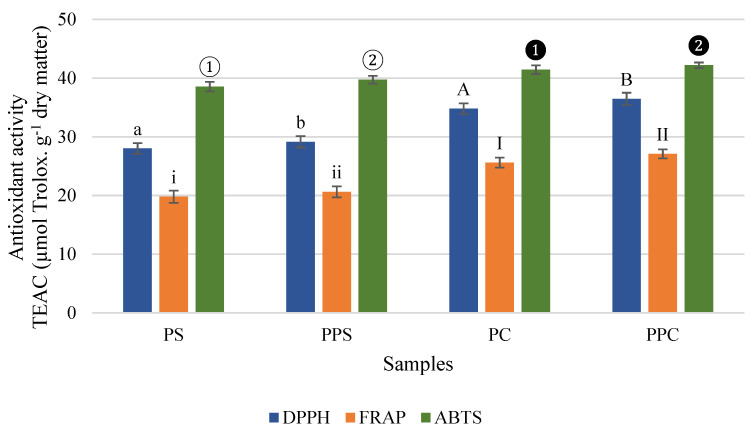
Antioxidant activity in pineapple shell (PS) and pineapple core (PC) samples, before and after pressurization (shell: PPS; core: PPC), by methods: (a) DPPH (2,2-diphenyl-1-picrylhydrazyl), (b) FRAP (Ferric Reducing Antioxidant Power), and (c) ABTS (2,2′-azino-bis(3-ethylbenzothiazoline-6-sulphonic acid)). The results were expressed by Trolox Equivalent Antioxidant Capacity (TEAC). Error bars represent ± standard deviation (n = 9). Statistical analysis express significant differences between pineapple shell and pressurized pineapple shell for DPPH (lowercase letters), FRAP (lowercase roman numeral) and ABTS (white ordinal number), and between pineapple core and pressurized pineapple core for DPPH (capital letters), FRAP (capital roman numeral), and ABTS (black ordinal number).

Regarding the antioxidant activity, evaluated by DPPH, FRAP, and ABTS methods (Figure 2), it is possible to verify that the PC has higher values for DPPH (34.80–36.45 µmol Trolox.g^−1^ dry matter), FRAP (25.60–27.09 µmol Trolox.g^−1^ dry matter) and ABTS (41.43–42.19 µmol Trolox.g^−1^ dry matter) than the PS for DPPH (28.01–29.15 µmol Trolox.g^−1^ dry matter), FRAP (19.79–20.62 µmol Trolox.g^−1^ dry matter), and ABTS (38.55–39.73 µmol Trolox.g^−1^ dry matter). These results are in agreement with the results obtained for PS and core juices by Campos et al. (2020) [29]. The antioxidant activity of the PPS and PPC samples showed higher values than the antioxidant activity of the non-pressurized shell samples by the DPPH, FRAP, and ABTS methods. The pressure treatment in the PS promoted a significant increase (*p* < 0.05) of 4% (DPPH and FRAP) and 3% (ABTS) in the antioxidant activity and in PC, the pressure treatment promoted a significant enhancement (*p* < 0.05) 5, 6, and 2% by the DPPH, FRAP, and ABTS methods, respectively (Figure 2).

The increase promoted by abiotic stress in antioxidant activity was less than the observed by Santos et al. (2020) [11]. The antioxidant activity for PS and PC samples obtained in a present study agree with the results for DPPH (shell: 20.43–35.01 µmol Trolox.g^−1^ dry matter; core: 33.94–41.34 µmol Trolox.g^−1^ dry matter), FRAP (shell: 23.84–39.85 µmol Trolox.g^−1^ dry matter; core: 24.17–34.76 µmol Trolox. g^−1^ dry matter) and ABTS (shell: 28.67–48.78 µmol Trolox.g^−1^ dry matter; core: 30.87–48.32 µmol Trolox.g^−1^ dry matter).

Pressurization implies a significant increase in antioxidant activity. The use of abiotic stresses by technologies such as wounding, heat treatment, and UV radiation to improve phenolic compounds and antioxidant activity has been studied in other foods, namely in carrots [41,42]. A possible explanation for the lower values in the present study may be the shorter time between the cutting of the fruits and the processing of the by-products by hydrostatic pressure. The time between cutting and processing influences enzymatic activity and is reflected in the response of enzymes to stress. The determination of antioxidant activity in PBP is still poorly studied, some studies have used different extraction methods and reported in different units, which does not allow the comparison of values.

The characterization of the pineapple fruit is more frequently performed than that of PBP. The pineapple extracted with ethanol yielded antioxidant activity values (dry mass) of 1.7 ± 0.19 µmol Trolox equivalent. g^−1^, 2.5 ± 0.0.13 µmol Trolox equivalent. g^−1^ and 1.7 ± 0.20 µmol Trolox equivalent. g^−1^ for the DPPH, FRAP, and ABTS methods, respectively. In case of the same samples extracted with methanol: acetone, the antioxidant activity values were 4.8 ± 0.10 µmol Trolox equivalent.g^−1^, 6.2 ± 0.26 µmol Trolox equivalent.g^−1^ and 7.7 ± 0.90 µmol Trolox equivalent.g^−1^ (dry mass) for the DPPH, FRAP, and ABTS methods, respectively [25]. A study carried out with 26 pineapple genotypes from China obtained mean values (fresh weight) of antioxidant activity by the DPPH methods of 12.20 µmol Trolox equivalent. g^−1^ and ABTS of 8.35 µmol Trolox equivalent. g^−1^ [43]. In another study, the antioxidant activity of pineapples was determined by the DPPH method and quantified 5.03 µmol Trolox.g^−1^ sample, dry weight basis [44]. Considering the studies mentioned above, it can be concluded that exists some typical variation of antioxidant activity of these samples, not only because of the variability of the biomass but the differences in the methods used. Another study also carried out with pineapple fruit obtained antioxidant activity of 53.42 µmol Trolox.g^−1^ sample (dry matter) by the DPPH method [45]. In general, the values of antioxidant activity obtained in the samples of pineapple fruits are lower than the values obtained for the antioxidant activity in the present study for the PBP (shell and core). The PBP presented attractive compounds that make their recovery very interesting.

### 2.4. Bromelain Activity

**Figure 3:**
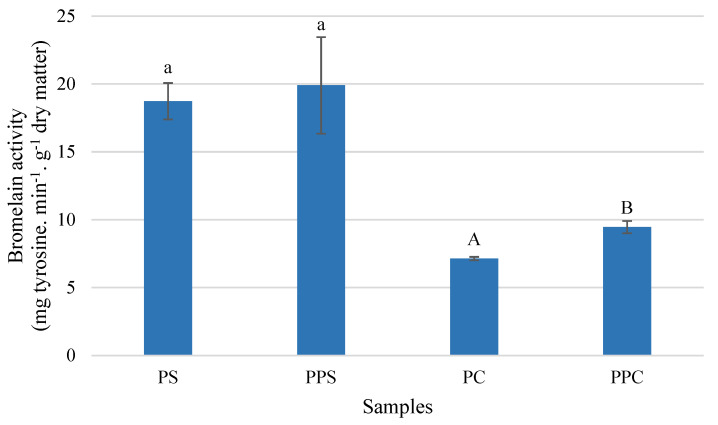
Bromelain activity in pineapple shell (PS) and pineapple core (PC) samples before and after pressurization (shell: PPS; core: PPC). Error bars represent ± standard deviation (n = 9). Different letters express significant differences between pineapple shell (lowercase letters) and pineapple core samples (capital letters).

As can be seen in Figure 3, bromelain activity is higher in the PS samples (18.73–19.90 mg tyrosine.min^−1^·g^−1^ dry matter) than in the PC samples (7.14–9.45 mg tyrosine.min^−1^·g^−1^ dry matter). The results obtained are in accordance with the range of values determined for PS (15.71–27.65 mg tyrosine.min^−1^·g^−1^ dry matter) and PC (5.09–16.81 mg tyrosine.min^−1^·g^−1^ dry matter) samples developed by Santos et al. (2020) [11].

The effect of pressurization on the bromelain activity, very evident in the study conducted by Santos et al. (2020) [11] (134% for PS and 350% for PC), was less marked in the present study, despite being positive. Bromelain activity increased for PPS (6%) and PPC (32%) samples, compared to untreated PS and PC samples, respectively. The effect of hydrostatic pressure on bromelain activity is still poorly studied and further studies are needed. The time and temperature at which the by-products are subjected after minimal processing of the fruit and before pressurization must be optimized. Some studies have been developed through the application of other abiotic stresses in vegetables and its effect on enzymatic activity (increase in PAL enzyme), and consequently in total phenolic compounds and antioxidant activity [39].

Given that bromelain is commonly ingested in the form of a pill or capsule (134.33 ± 1.34 mg tyrosine.min^−1^·g^−1^ dry matter), the ingestion of 7 g of dehydrated PS by-products allows obtaining the amount of bromelain equivalent to a pill. In the case of dehydrated PC, it would be necessary to consume a double approach. Ingesting PBP could suppress the need for bromelain pills to treat some diseases or promote health.

### 2.5. Microbiology (Aerobic Colony Counts, Yeasts, and Moulds)

The results obtained for the enumeration of microorganisms in the samples are shown in Table 3. The PS showed levels of mold considered unsatisfactory (>10^3^ CFU·g^−1^) for fruit products [46], both in raw samples as pressurized. However, the PC samples showed satisfactory values for the three quality criteria analyzed, although also regardless of the pressurization treatment. As a suggestion to reduce the levels of mold in the PS, it would be interesting to intensify or improve the washing and disinfection of pineapple fruits before cutting in the minimally processed industry.

### 2.6. Mycotoxins

The quantification of mycotoxins aflatoxin B_1_, aflatoxin B_2_, aflatoxin G_1_, aflatoxin G_2_, ochratoxin A, and patulin in PS and PC can be seen in Table 4.

The PC samples showed results below the detection limit of the method for aflatoxins and ochratoxin A. Some toxins were detected (aflatoxin B_2_, aflatoxin G_1_, and ochratoxin A) in the PS samples, however, in very low values. All samples (PS and PC) showed results below the detection limits of the patulin method (2.9 µg·kg^−1^). The recovery rates of the analytical method are also found in the analytical performance requirements (between 50–110%).

Current legislation does not apply to the quantification of toxins in PBP. The results are much lower than what the legislation allows for the occurrence in infant feeding, considering the valorization and interpretation of the results based on the regulation CE 1811/2006 [47].

The inhibition of mycotoxin development may be related to the acidity of the sample (3.62 ± 0.02 to 3.65 ± 0.04), as observed in other studies [48]. The refrigeration temperature conditions before treatment of hydrostatic pressure and the water activity during the storage are also important factors in limiting mycotoxin biosynthesis [49].

## 3. Materials and Methods

### 3.1. Sample Preparation

Pineapple (*Ananas comosus* L.) by-products (10 kg) were supplied by the company Campotec S.A. located in Torres Vedras, Portugal. The 5 kg pineapple shell (~110 × 40 mm) and 5 kg pineapple core (~104.5 × 30 mm) were stored under refrigeration (5 ± 1 °C) approximately 3 h prior to packaging in PA/PE-90 (Alempack - Embalagens Flexíveis, Elvas, Portugal) that were vacuum sealed (85% of vacuum).

Hydrostatic pressure treatment as an abiotic stress was applied in packaged by-products (shell and core of pineapple) using a pilot-scale high-pressure hydrostatic equipment (Hiperbaric 55, Burgos, Spain) with a 55 L vessel, according to the conditions optimized in a previous work (225 MPa during 8.5 min) [11]. PBP samples after pressurization were stored at 5 ± 1 °C for 24 h. After hydrostatic pressure stress, enzymes need time for the development of metabolic reactions that produce the compounds of interest. Following this time, all samples were frozen at −80 °C and, posteriorly lyophilized (Coolsafe Superior Touch, Scanvac, Denmark). After that, samples (300 g) were wound in a food processor (Thermomix TM31, Vorwerk Thermomix, Wuppertal, Germany) at speed 10 (~10,200 rpm) for 25 s, to obtain the powder sample. Control samples were not subjected to hydrostatic pressure treatment.

### 3.2. Analytical Methods

The PS and PC by-products samples (raw and after high-pressure treatment) were used for the analysis of: moisture; water activity; caloric value; protein; carbohydrates; ash; minerals; soluble, and insoluble fiber; total phenolic content; antioxidant activity (DPPH, FRAP, and ABTS); bromelain activity; microbial content (total mesophilic microorganisms, yeasts, and molds); and mycotoxins (aflatoxin B_1_, aflatoxin B_2_, aflatoxin G_1_, aflatoxin G_2_, ochratoxin A, and patulin).

#### 3.2.1. Color

The color was evaluated on the surface of PBP powder with a Minolta CR 300 (Konica Minolta, Osaka, Japan) using the L*, a*, b* coordinates (CIELab color system). The calibration was performed with a white ceramic reference (standard illuminant D65). The color was evaluated ten times on each pineapple by-product sample and the color parameter value resulted from the arithmetic mean of ten measurements.

#### 3.2.2. Moisture Content and Water Activity (a_w_)

The water content of the samples was determined gravimetrically by mass loss in an oven at 105 ± 1 °C until constant weight, in three replicates. Water activity was determined using a Rotronic HygroPalm HP23-A equipment, Bassersdorf, Switzerland (20 °C).

#### 3.2.3. Caloric Value

Energy was calculated by the complete combustion of samples in an adiabatic calorimeter (Parr 1261; Parr Instrument Company, Moline, IL, USA) [50].

#### 3.2.4. Protein

Protein analysis was performed by the Kjeldhal method using equipment with a semi-automatic Kjeltec system, Effretikon (Zurich), Switzerland, according to the AOAC 920.152 official method for fruit products [51]. The determination of crude protein occurred in three phases. The first was digestion, 1 g of sample was added with kjeltab and sulfuric acid, and then it was placed in the digestion unit at 400 °C for 60 min. The second step was distillation; the solution was placed in the distillation unit that added distilled water and NaOH solution (50%). The distillate was collected in 1% boric acid with an indicator, and titration was performed with 0.1 mol·L^−1^ HCl solution. The protein determination was performed in triplicate.

#### 3.2.5. Carbohydrates

The determination of total carbohydrates was performed by the phenol-sulphuric acid method with some modifications [52]. Briefly, the samples (50 mg) were reconstituted with distilled water [28] and extracted four times with 10 mL ethanol (80%) in sealed tubes by boiling at 95 °C for 10 min in a water bath. After each extraction, the tubes were centrifuged at 2500 rpm for 5 min, and the supernatants of the four extractions were combined. The extract samples were mixed with phenol (5%) and concentrated sulfuric acid. The color was developed after 10 min in a dark place and 20 min of cooling in a water bath at 25–30 °C. The absorbance was measured at 490 nm against a reagent blank on a spectrophotometer. The total carbohydrates were defined by a standard curve of D-glucose. Each sample was replicated six times.

#### 3.2.6. Dietary Fiber

The determination of the total, soluble and insoluble dietary fiber contents of PS and PC were carried out according to the AOAC 991.43 method [53]. Dietary fiber was analyzed using the fiber assay kit (Megazyme K-TDFR, Wicklow, Ireland). In the PC samples, the sugar was extracted before determining the dietary fiber. The sample extraction (10 mL·g^−1^) was performed with 85% ethanol, centrifuged at 8000 rpm for 20 min, and the pellet was collected. This process was repeated three times, and subsequently, the sample was dried overnight at 40 °C. The content of the dietary fiber was corrected for residual protein (Kjeldahl method), ash, and white. The PC samples were also corrected for the sugar fraction removed. The fiber was performed six times and the average was used for each condition.

#### 3.2.7. Ash Content

Ash content was determined gravimetrically by incineration at 550 °C in a muffle furnace (Heraeus 260E, Katzwinkel, Germany) according to the AOAC 940.26 method [51]. The ash was carried out in triplicate and the average was used for each condition.

#### 3.2.8. Minerals

The mineral content was evaluated by inductively coupled plasma spectrometry (ICP) (iCAP Spectrometer equipped with ASX-520 AutoSampler (Thermo Scientific, Waltham, MA, USA)). The lyophilized sample (0.25 g) was transferred to digestion vessels, 9 mL of HCl and 3 mL of nitric acid (HNO_3_) were added. Digestion took place in several stages: (1) 30 min/40 °C, (2) 30 min/80 °C, and (3) 90 min/105 °C in the SCP Science equipment (DigiPREP MS, Baie d’Urfe, QC, Canada).

After cooling, 50 mL of distilled water was added before being decanted. The cleared supernatant was used in inductively coupled plasma to determine the elements (sodium, potassium, calcium, magnesium, phosphorus, sulfur, iron, copper, zinc, manganese, and boron). The analyses were repeated in triplicate.

#### 3.2.9. Pineapple Extract Preparation for Total Phenolic Content and Antioxidant Activity

The PBP extract was prepared by the procedure of Heredia and Cisneros-Zevallos (2009) and Swain and Hillis (1959) [54,55]. The extraction was performed in a ratio of 1:10 (*w*:*v*) of three PBP independent samples and methanol (100%) followed by Ultra-Turrax homogenizer at 8000 rpm for 2 min. The homogenates were incubated overnight (12–24 h) at 4 °C. Subsequently, the extracts were centrifuged at 8000 rpm for 20 min (4 °C), and the supernatants were stored at 4 °C, protected from light until analysis.

##### Total Phenolic Content (TPC)

The total phenolic content (TPC) was determined according to Heredia and Cisneros-Zevallos (2009), and Swain and Hillis (1959) [54,55]. The extract aliquots (150 µL) were diluted with 2400 μL nanopure water, mixed with 150 µL Folin-Ciocalteu reagent (Panreac AppliChem, Germany) (0.25 mol·L^−1^). The reaction was interrupted by adding 300 µL sodium carbonate (1 mol·L^−1^) and the mixture was incubated (2 h). The samples were read at 725 nm in a spectrophotometer (UNICAM UV/Vis Spectrometer, United Kingdom). The total phenolics content was determined by a standard curve of chlorogenic acid equivalents (CAE) and expressed as mg CAE·g^−1^ dry weight. Each extract was analyzed in triplicate and the average was used for each condition.

##### Antioxidant Activity (DPPH, FRAP, and ABTS)

The antioxidant capacity by DPPH (2,2-diphenyl-1-picrylhydrazyl) method was evaluated following the procedure of Brand-Williams et al. (1995) [56]. The DPPH solution was prepared with DPPH reagent (Sigma–Aldrich, Darmstadt, Germany) diluted in methanol until reaching 1.1 units of absorbance at 515 nm. The supernatant (100 μL) was combined with 3.9 mL DPPH solution, and the reaction occurred for 40 min. The samples were read at 515 nm using a spectrophotometer (UNICAM UV/Vis Spectrometer).

The FRAP (ferric reducing antioxidant power) test was performed according to Benzie and Strain (1996) [57]. The reaction initiated with the mixture of the FRAP solution (2.7 mL), 270 μL nanopure water with the extract samples (90 μL), and afterwards, warmed in a water bath at 37 °C for 30 min. The colored product (ferrous tripyridyltriazine complex) was read at 595 nm.

Antioxidant activity was also measured using ABTS (2,2′-azino-bis(3-ethylbenzothiazoline-6-sulphonic acid)) method as described by Re et al. (1999) and Rufino et al. (2007) [58,59]. The reaction was performed by mixing 2970 μL ABTS solution (Sigma–Aldrich, Germany) with sample aliquots (30 μL) for 6 min, and the absorbance at 734 nm was read. The antioxidant activity (DPPH, FRAP e ABTS) was determined using Trolox (Acros Organics, Belgium), and the results were expressed by Trolox Equivalent Antioxidant Capacity [TEAC (µmol Trolox.g^−1^ dry matter)]. For each antioxidant determination method (DPPH, FRAP, and ABTS), each extract was analyzed in triplicate and the average was used for each condition.

#### 3.2.10. Bromelain Activity

The bromelain assay was established according to Chakraborty et al. (2014) [60] with some modifications. The reaction involved 50 μL enzyme extract (from PBP) and 1150 μL casein 1% (*w*/*v*) (Sigma–Aldrich, Germany) solution in glycine 0.1 mol·L^−1^ (Sigma–Aldrich, Germany) and 25 mmol·L^−1^ cysteine (Sigma–Aldrich, Germany). The mixture was incubated in a shaking water bath (10 min at 37 °C) and the reaction was stopped by adding 1.8 mL trichloroacetic acid 5% (*w*/*v*). The assay mixture was filtrated with a syringe filter (0.45 μm) and the absorbance measurement was performed at 280 nm. The bromelain activity was calculated using a tyrosine (Alfa Aesar, Lancashire, UK) standard curve and expressed as the amount of tyrosine on a dry weight basis reported as μmol tyrosine. min^−1^·g^−1^ dry weight.

#### 3.2.11. Microbiological Analysis (Aerobic Colony Count, Yeasts and Molds)

For the enumeration of mesophiles (aerobic colony count) of yeasts and molds, 10 g of each sample was aseptically weighed into sterile BagFilters (Interscience, France), and 90 mL of Ringer Solution (RS) was added. Samples were homogenized using a paddle blender, 85 rpm for 30 s. Afterwards, decimal dilutions were made with RS diluent according to ISO Standard [61]. The aerobic colony count was performed according to ISO 4833:2003 [62], incorporating 1 mL of the respective decimal dilution in Plate Count Agar medium (Biokar Diagnostics, Beauvais, France) and incubating for 72 ± 3 h at 30 ± 1 °C.

Enumeration of molds and yeasts was carried out based on ISO 21527-2:2008 [63], by spreading 0.1 mL of the respective decimal dilution onto plates of Glucose Yeast Peptone Agar (GYP). Subsequently, the plates were incubated at (25 ± 1) °C for 5 days before counting the colonies (CFU) of molds and yeasts per gram of sample.

#### 3.2.12. Mycotoxins

##### Aflatoxins and Ochratoxin A

The extraction of aflatoxins (B_1_, B_2_, G_1_, and G_2_) and ochratoxin A in samples of PBP was carried out with 10 g of sample and 50 mL of methanol/water (80:20, *v*/*v*). The extract was filtered using folded filter paper and a 10 mL aliquot was diluted with phosphate-buffered saline to 100 mL, filtered once again with a glass microfiber filter, and introduced in an immunoaffinity column containing specific antibodies to aflatoxins and ochratoxin A (AflaOchra, Vicam). The elution was carried out with 1.6 mL methanol (HPLC grade), with successive dilution with water to 10 mL (16:84, *v*/*v*). A volume (800 µL) was injected into the HPLC system.

HPLC analysis was performed using a Waters Alliance 2695 equipment with a Waters 2475 fluorescence detector (Waters, Milford, MA, USA) with Empower Chromatography Software. Post-column derivatization was performed with electrochemically generated bromine (Kobra cell, R-Biopharm). The chromatographic column was a Prodigy ODS 100 Ǻ (5 µm, 150 × 4.6 mm, Phenomenex, Torrance, CA, USA). The mobile phases were a gradient involved with a flow rate of 1 mL·min^−1^ between phase A [KBr (175 mg·L^−1^)/MeOH/MeCN/C_2_H_4_O_2_ (1650:465:390:50)] and phase B [KBr (175 mg·L^−1^)/MeOH/MeCN/C2H4O2 (140:1283:1073:50)]. The temperatures of the automatic sampler were maintained at 10 °C and the temperature of the chromatography column at 35 °C. The total run time was 59 min. The wavelength of the fluorescence detector was 365 and 322 nm (excitation), and 465 and 468 nm (emission) for aflatoxins and ochratoxin A, respectively (Martins et al., 2018; Sizoo and Van Egmond, 2005) [64,65].

##### Patulin

Stock solution with 200 mg·L^−1^ of patulin (Sigma–Aldrich) was prepared in ethyl acetate, stored at −20 °C, and when required, some volume was evaporated and diluted with ethanol for the formulation of an intermediate standard solution. The patulin intermediate standard concentration solution was determined by UV at 276 nm, compared to a solvent blank, applying the molar extinction value. The intermediate standard solutions were diluted with acetic acid (0.1%) with the aim of develop final concentrations between 2 and 200 µg·L^−1^.

The HPLC equipment was a Waters Alliance 2695 system equipped with a photodiode array detector (Waters 2996), set at 276 nm and with a 2000 µL loop. Data compilation and following processing were performed utilizing the Empower II software. A stainless steel analytical column (250 × 4.6 mm i.d., 4 µm, SynergyHydro-RP C18; Phenomenex, Tecnocroma, Portugal) preceded by a guard column (4 × 3 mm i.d.) with the equal stationary phase was applied. The mobile phase, eluting at a flow rate of 1 mL·min^−1^, contained an isocratic blend of water–acetonitrile–perchloric acid (96:4:0.1) for 20 min followed by a gradient washing (5 min) stage, which begins with a concentration of acetonitrile (100%) and closes with acetonitrile (65%) in water. Two hundred microliters were injected onto the HPLC column.

Patulin was extracted from the samples of PBP using ethyl acetate solvent in the presence of sodium sulfate and sodium hydrogen carbonate. The extracts were cleaned (or purified) using a solid-phase extraction of unconditioned silica gel (SPE) column (Strata Si 500 mg/3 mL, Phenomenex, Tecnochroma, Portugal). The purified extract was evaporated, dissolved again in aqueous acetic acid solution (pH 4), separated by reversed-phase (RP) HPLC, and quantitatively determined by UV detection at 276 nm. The redissolved samples were filtered with a membrane filter (Millex 0.45 µm, Millipore) and the filtrate transferred to HPLC flasks. The samples were analyzed in duplicate and a third sample enriched with patulin at 20 µg·kg^−1^. The identification of the patulin peak of the sample extracts was produced by comparison with the retention time of standard solutions and samples fortified with patulin. Additional validation of patulin was achieved by comparing peak spectra with standard spectra found under the same chromatographic requirements and peak purity assessment [66,67].

### 3.3. Data Interpretation and Analysis

For each sample submitted to microbiological analysis, average values and respective mean difference from duplicate plates were calculated. The guidelines for the microbiological quality of ready-to-eat foods sampled at the point of sale of the National Institute of Health, Dr. Ricardo Jorge, Portuguese Ministry of Health [46] were used for the interpretation of the microbiological quality of the pineapple samples. Physicochemical and enzymatic characterization data were statistically evaluated through the Statistica^TM^ v.8 Software (StatSoft Inc., Tulsa, OK, USA 2007) from Statsoft (StatSoft Inc., Tulsa, OK, USA) [68]. Tukey’s honest significant difference (HSD) test was applied to determine the significant differences among means for different treatments. The accepted level of significant differences was *p* < 0.05.

## 4. Conclusions

The dehydrated pineapple by-products (shell and core) from the minimally processed industry showed significant levels of value-added compounds, namely dietary fiber, minerals, and phenols, and high values of bromelain activity. Abiotic stresses by moderate hydrostatic pressure were demonstrated to be beneficial in enhancing total phenolic content, antioxidant activity, and bromelain activity without affecting the nutritional value. The microbiological and mycotoxins results show that the core by-product have adequate characteristics to be used as food ingredients, however, the shell needs to be subjected to more efficient prior decontamination.

In conclusion, the re-incorporation of dehydrated pineapple core into the food chain as an ingredient rich in antioxidants and bromelain is of great interest, leading to a virtuous cycle of waste reduction, optimizing the use of natural resources and the preservation of the ecosystems, and creating value.

## Figures and Tables

**Table 1:** The color of pineapple shell samples and pineapple core samples: L*, a* and b* CIE L*a*b* color parameters, and photography. Mean values ± standard error, n = 10. Different letters express significant differences between the color of pineapple shell (PS) and pressurized pineapple shell (PPS) (lowercase letters), and pineapple core (PC) and pressurized pineapple core (PPC) samples (capital letters).

Color	PS	PPS	PC	PPC
L*	79.91 ± 1.02 ^a^	79.49 ± 0.83 ^a^	90.44 ± 0.34 ^A^	89.44 ± 1.61 ^A^
a*	−2.61 ± 0.36 ^a^	−2.07 ± 0.16 ^b^	−1.94 ± 0.13 ^A^	−2.09 ± 0.23 ^A^
b*	25.46 ± 0.36 ^a^	25.99 ± 0.97 ^a^	23.22 ± 0.56 ^A^	24.80 ± 0.77 ^B^
**Photography**	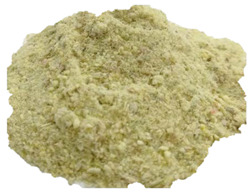		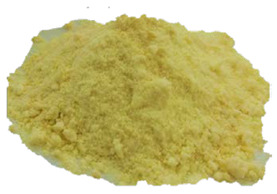	

**Table 2:** Chemical and physical characterization of the dehydrated samples: pineapple shell (PS) and pineapple core (PC). Different letters express significant differences between pineapple shell and pineapple core samples. Mean values ± standard error. Dietary fiber (n = 6), other parameters (n = 3).

			PS	PC
**Moisture** (g.100 g^−1^)			5.50 ± 0.28 ^a^	7.96 ± 0.17 ^b^
**Water activity**			0.264 ± 0.004 ^a^	0.299 ± 0.011 ^b^
**Caloric value** (kcal.100 g^−1^ dry matter)			374.52 ± 1.83 ^b^	363.89 ± 3.90 ^a^
**Protein**	(g.100 g^−1^ dry matter)		3.86 ± 0.10 ^b^	2.71 ± 0.20 ^a^
**Carbohydrates**			66.44 ± 6.29 ^a^	87.58 ± 3.37 ^b^
**Ash**			4.23 ± 0.13 ^a^	4.83 ± 0.28 ^b^
**Dietary fiber**		Insoluble	28.82 ± 1.58 ^b^	16.17 ± 1.20 ^a^
		Soluble	3.63 ± 0.13 ^b^	1.53 ± 0.46 ^a^
		Total	32.42 ± 1.55 ^b^	17.70 ± 1.45 ^a^
**Minerals**	(mg·kg^−1^ dry matter)	Sodium	277.87 ± 22.07 ^a^	266.24 ± 8.55 ^a^
		Potassium	9997.69 ± 108.13 ^a^	12089.92 ± 93.91 ^b^
		Calcium	1592.45 ± 80.18 ^b^	375.65 ± 43.22 ^a^
		Magnesium	784.71 ± 13.87 ^a^	964.45 ± 11.71 ^b^
		Phosphorus	1552.87 ± 8.37 ^b^	545.17 ± 8.84 ^a^
		Sulphur	1183.92 ± 19.18 ^b^	601.98 ± 3.48 ^a^
		Iron	32.79 ± 2.84 ^b^	19.44 ± 0.68 ^a^
		Copper	5.16 ± 0.78 ^a^	5.80 ± 0.34 ^a^
		Zinc	8.91 ± 0.04 ^b^	1.81 ± 0.05 ^a^
		Manganese	34.97 ± 1.06 ^b^	21.90 ± 0.59 ^a^
		Boron	6.66 ± 0.66 ^b^	2.75 ± 0.37 ^a^

**Table 3:** Microbiological characterization of the samples (aerobic colony count, mold and yeast enumeration) in the samples: pineapple shell (PS), pressurized pineapple shell (PPS), pineapple core (PC), and pressurized pineapple core (PPC). Mean values ± mean difference, n = 2.

Samples	Aerobic Colony Count (CFU·g^−1^)	Mould Count (CFU·g^−1^)	Yeast Count (CFU·g^−1^)
**PS**	4.10 (±2.0) × 10^4^	2.30 (±0.1) × 10^4^	3.85 (±1.6) × 10^4^
**PPS**	3.25 (±0.2) × 10^4^	5.25 (±0.1) × 10^4^	1.50 (±0.1) × 10^3^
**PC**	1.90 (±0.6) × 10^2^	2.00 (±0.1) × 10^2^	1 (±0.0) × 10^2^
**PPC**	1.20 (±0.1) × 10^2^	2.00 (±0.0) × 10^2^	<10^2^
**Microbiological quality guidelines** [46]	**Satisfactory < 10^6^**	**Satisfactory < 5 × 10^2^** **Borderline 5 × 10^2^ < 10^3^** **Unsatisfactory > 10^3^**	**Satisfactory < 10^5^**

**Table 4:** Results of mycotoxins quantification (aflatoxin B_1_, aflatoxin B_2_, aflatoxin G_1_, aflatoxin G_2_, ochratoxin A, and patulin) in the samples: pineapple shell (PS), pressurized pineapple shell (PPS), pineapple core (PC), and pressurized pineapple core (PPC). * Corrected results for the recovery rate; DL, detection limit; QL, quantification limit.

Samples	Mycotoxin results	Aflatoxin B_1_	Aflatoxin B_2_	Aflatoxin G_1_	Aflatoxin G_2_	Ochratoxin A	Patulin
**PS**	Result (µg/kg)	<DL	0.008 *	0.033 *	<DL	<DL	<DL
	Recovery Rate (%)	81	83	77	67	89	89
**PPS**	Result (µg/kg)	<DL	<DL	0.013 *	<DL	0.051 *	<DL
	Recovery Rate (%)	81	104	79	66	79	74
**PC**	Result (µg/kg)	<DL	<DL	<DL	<DL	<DL	<DL
	Recovery Rate (%)	105	106	104	81	68	62
**PPC**	Result (µg/kg)	<DL	<DL	<DL	<DL	<DL	<DL
	Recovery Rate (%)	94	95	92	73	65	51
**Reference values**	Detection limit, DL (µg/kg)	0.011	0.004	0.007	0.004	0.027	2.9
	Quantification limit, QL (µg/kg)	0.038	0.013	0.023	0.014	0.089	7.4

## Data Availability

Not applicable.

## Sample Availability

Not available.
